# The many roads to and from multicellularity

**DOI:** 10.1093/jxb/erz547

**Published:** 2019-12-10

**Authors:** Karl J Niklas, Stuart A Newman

**Affiliations:** 1 Plant Biology Section, School of Integrative Plant Science, Cornell University, Ithaca, NY, USA; 2 Department of Cell Biology and Anatomy, New York Medical College, Valhalla, NY, USA; 3 University of Essex, UK

**Keywords:** Algae, evolution, fungi, land plants, metazoans, multilevel selection theory, unicellular bottleneck, *Volvox*

## Abstract

The multiple origins of multicellularity had far-reaching consequences ranging from the appearance of phenotypically complex life-forms to their effects on Earth’s aquatic and terrestrial ecosystems. Yet, many important questions remain. For example, do all lineages and clades share an ancestral developmental predisposition for multicellularity emerging from genomic and biophysical motifs shared from a last common ancestor, or are the multiple origins of multicellularity truly independent evolutionary events? In this review, we highlight recent developments and pitfalls in understanding the evolution of multicellularity with an emphasis on plants (here defined broadly to include the polyphyletic algae), but also draw upon insights from animals and their holozoan relatives, fungi and amoebozoans. Based on our review, we conclude that the evolution of multicellular organisms requires three phases (*origination* by disparate cell–cell attachment modalities, followed by *integration* by lineage-specific physiological mechanisms, and *autonomization* by natural selection) that have been achieved differently in different lineages.

## Teaser

The evolution of multicellular organisms, which requires three phases (origination, integration, and autonomization), has been achieved differently in different lineages.

## Introduction

The evolution of multicellularity has been conceptualized as a major evolutionary transition (Maynard Smith and Szathmáry, 1995; Bonner, 1998). Estimates of the exact number of independent events differ depending on how multicellularity is defined. When broadly defined as the ability to sustain cellular congeries, estimates indicate that eukaryotic multicellularity evolved over 25 times (Grosberg and Strathmann, 2007). However, when restricted to *intrinsically multicellular organisms* (IMOs, i.e. those with cell to cell signaling and a heritable phenotype), estimates narrow down to only 11 occurrences among eukaryotes—once in the Amoebozoa (dictyostelids), once in the Animalia or Metazoa, three in the Fungi (chytrids, ascomycetes, and basidiomycetes), and twice in each of the three major photosynthetic eukaryotic clades (Niklas and Newman, 2013; Niklas, 2014). It also evolved (and has been lost) among the prokaryotes, e.g. phylogenetic analyses using 16S rDNA sequences indicate that most of the morphological diversity observed among extant cyanobacteria, including the majority of single-celled species, likely evolved from ancient multicellular lineages, and that the multicellular phenotype was regained at least once after a previous loss (Schirrmeister *et al.*, 2011).

Regardless of how multicellularity is defined or how many times it evolved, its multiple origins raise a number of important, but largely unanswered, questions. What (if any) are the selection factors favoring its appearance? Are the recurrent phenotypic motifs that appear in multicellular lineages the result of adaptive evolution, or are they the unavoidable consequences of simple biophysical processes? Equally compelling questions concern the genomic regulation of multicellularity, e.g. do all multicellular lineages share similar genomic regulatory motifs, and, if so, how do they operate? The answers to these and related questions have far-reaching consequences, e.g. tumorigenesis and neoplasm growth are posited to result from the disruption of molecular networks established in the unicellular to multicellular transition (reviewed by Trigos *et al.*, 2018).

Recent research focusing on colonial and multicellular organisms has shed light on these questions, particularly the genomic and developmental origins and regulation of multicellularity (Box 1). Notable among recent achievements is the use of artificial selection regimes to induce simple multicellularity (Ratcliff *et al.*, 2013; Coates *et al.*, 2014; Herron *et al.*, 2019), and genomic profiling of large-scale changes in gene-expression patterns within multicellular life cycles (Prochnik *et al.*, 2010; Hanschen *et al.*, 2016; Herron *et al.*, 2018; Krizsán *et al.*, 2019). Other advances are the recognition that the last eukaryotic common ancestor (LECA) to all eukaryotic lineages was likely considerably more complex than previously thought. For example, the LECA likely had a genome encoding the endomembrane apparatus, spliceosome, nuclear pore, myosin and kinesin cytoskeletal motors, and a ubiquitin signaling system, and there is evidence for the parallel deployment of ancient regulatory DNA-binding domains (e.g. the SAND DNA-binding proteins) in widely divergent lineages (e.g. green algae, land plants, and animals) (Young *et al.*, 2011; Grau-Bové *et al.*, 2017; Nedelcu, 2019). The discovery of new organisms has also resulted in key insights, e.g. the discovery of the colonial choanoflagellate *Choanoeca flexa* has shown that the capacity for collective cell constriction—a feature common in most animal lineages—likely preceded the evolution of metazoans (Brunet *et al.*, 2019).

Box 1. **Key recent developments in understanding the evolution of multicellularity**

- Large-scale changes in lineage-specific and phyletically shared gene expression patterns underlie the unicellular to multicellular life cycle transition.

Herron *et al.* (2018) report that dramatic changes in *Chlamydomonas*-specific and volvocine-specific cell cycle and reproductive gene–process expression patterns accompany the appearance of a multicellular life cycle derived from a unicellular one.

-  The SAND DNA binding domain functions in animals, green algae, and land plants.

Nedelcu (2019) reports that the SAND domain (a DNA-binding domain functioning in the regulation of cell differentiation and proliferation) is unique to animals and the viridiplantae, and suggests that its presence is best explained by a lateral gene transfer event from a green alga to an early metazoan.

-  Cell aggregation in a colonial green alga involves the co-option of the retinoblastoma cell cycle regulatory pathway.

Hanschen *et al.* (2016) show that expression of the retinoblastoma cell cycle regulatory pathway of a simple colonial, green alga (*Gonium pectoral*) in a unicellular green alga (*Chlamydomonas*) results in a colonial body plan demonstrating the co-option of cell cycle regulation during the evolution of multicellularity.

-  Complex animal multicellularity was preceded by dynamic genomic changes at the unicellular level.

By reconstructing the genomic machinery of pre-metazoans, Grau-Bové *et al.* (2017) reveal that multiple changes in gene diversity coupled with an ancient cell adhesion machinery occurred in the unicellular ancestor of the metazoans.

-   Cell-type diversity and its genome regulation in the holozoan relatives of animals indicates that the last unicellular ancestor of animals was capable of elaborate cell-type specification.

De Mendoza *et al.* (2015) show that the development of the ichthyosporean *Creolimax* involves the dynamic regulation of alternative splicing and long inter-genic non-coding RNAs similar to the animal cell-type specification machinery.

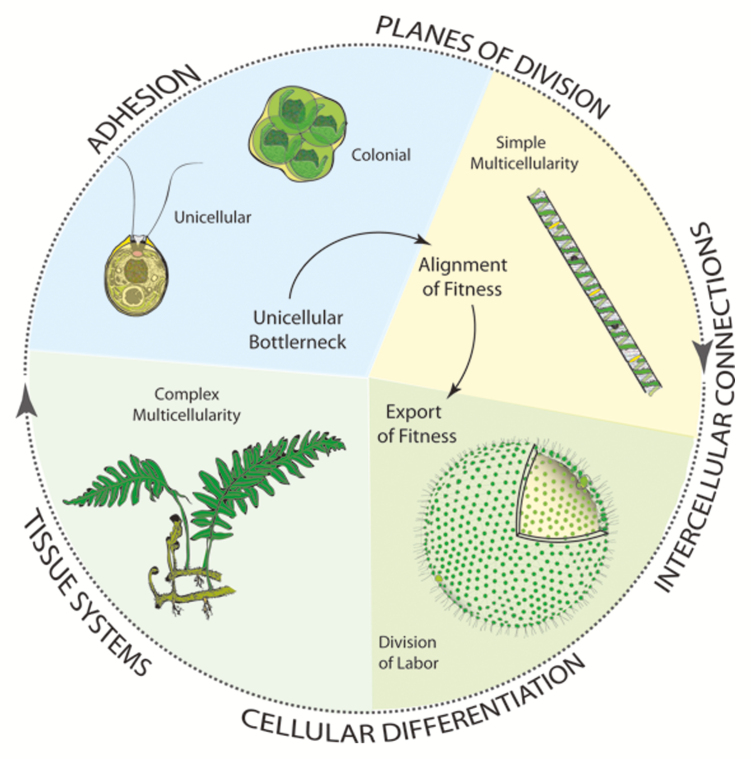

The standard model for a unicellular-to-multicellular transition involves a colonial body plan intermediate, a unicellular bottleneck, and an export of fitness from the cellular to the multicellular level of organization. The taxa shown here are not phylogenetically closely related and are used only to illustrate key innovations.

Further, evidence is mounting that a strict adaptationist narrative for the evolution of multicellularity is misleading in light of the capacity of simple biophysical processes to generate multicellular phenotypes, and the pronounced differences in the motifs required to achieve multicellularity among lineages. For example, all eukaryotic cells can secrete polysaccharides and structural glycoproteins that self-assemble to form extracellular matrices, which contain interpenetrating polymeric networks with hydroxyproline-rich glycoproteins as their major scaffolding components (e.g. the hydroxyproline-rich glycoprotein extensin superfamily in various algae and the land plants, and collagens in animals). Although the composition of algal and land plant cell walls manifest some similarities, as for example the presence of cellulose (Niklas, 2004), the adhesives used to bind cells together are biochemically vastly different among multicellular eukaryotes (e.g. carbohydrate and protein adhesives in land plants and metazoans, respectively). Likewise, standard multilevel selection theory for multicellularity posits that a unicellular ‘bottleneck’ is required to reduce intercellular conflict. Yet, even asexual life cycles can have such a bottleneck (e.g. cyanobacterial heterocysts and akinetes), and every life cycle involving a meiotic event inevitably has one. Indeed, the land plant life cycle has two (i.e. spores and zygotes; Niklas and Kutschera, 2010). Thus, unicellular bottlenecks may be exaptations conferring immunity to future cell–cell conflicts rather than being adaptations *per se*.

## Adaptationist models of multicellularity

The evolution of IMOs required a transition from unicellular forms to entities composed of interactive and collaborative cells. A key question concerning the dynamics of this transition is, what, if any, were the factors driving this transition—were they biotic or abiotic? Traditional answers take on an adaptationist perspective in which natural selection operating on unicellular variants within populations eliminates maladaptive elements to produce multicellular entities that are reproductively more fit. Although some form of *ad hoc* selection is necessary for any stable evolutionary transition (i.e. the persistence of a novelty depends on its reproductive success), an adaptationist perspective *sensu stricto* neglects the possibility that multicellularity may not confer an initial advantage, but rather reflect a simple physical sorting of cellular variants with differential adhesion. Such manifestations have been called ‘neutral phenotypes’ (Bonner, 2013; see also Newman, 2019*a*).

As noted, all unicellular organisms produce adhesives to cling to a substrate, engage in syngamy, etc. A differential sorting mechanism can in theory generate maladapted multicellular entities (e.g. non-motile aquatic photosynthetic organisms with a greater settling velocity would have diminished access to sunlight) that could subsequently obtain an advantage (e.g. diminished predation owing to their larger size). Such scenarios illustrate that an adaptationist perspective for the initial appearance of multicellularity is not *a priori* correct since unicellular elements in a population can drift into simple multicellular elements by means of simple physical processes operating via the functionality of biological adhesives.

Regardless of the factors preconditioning the appearance of multicellularity, the standard model(s) for multicellularity posit a unicellular to colonial to simple multicellular transition series. Although alternative scenarios are biologically feasible (Niklas *et al.*, 2013; Niklas, 2014), it is reasonable to speculate that the colonial body plan typically preceded the appearance of simple multicellularity. A variety of plausible models has been proposed to explain how the defining characteristics of complex multicellular organisms evolved (e.g. intercellular cooperation and cellular differentiation). Once again, most of these models, even those involving ‘cheating’, emphasize the role of natural selection. For example, experiments have shown that lineages of ‘cooperating’ bacteria propagated under strong selection rewarding the persistence of collectives produce life cycles that either purge cheating cells or reinforce their presence, and that the latter life cycles have alternating phenotypic states in which cheating cells function as propagules (Hammerschmidt *et al.*, 2014).

However, we draw attention to the neglect of simple physical processes, such as passive diffusion and metabolic phenomena inherent to all life forms. For example, cells within a colony lacking direct contact with their external fluid environment may experience an accumulation of metabolites (or experience reduced access to external resources) necessitating changes in their gene regulatory pathways, or in extreme cases resulting in apoptosis. Such changes can, in theory, drive the evolution of different cell types. Indeed, most unicellular organisms achieve different metabolic and morphological states during their life cycles. Consequently, cell differentiation during a colonial to multicellular transition need not result from natural selection *sensu stricto*, but rather reflect the co-option of ancestral traits for new functionalities (Kiss *et al.*, 2019; Newman, 2019*b*).

If an exaptation or neutral phenotype perspective is adopted as being no less plausible than an adaptationist perspective, the loss of multicellularity and the many examples of reduction in multicellular complexity become easier to explain. Just as there are advantages to being large and morphologically complex, there are many advantages to being small and morphologically simple, e.g. unicellular and small multicellular photoautotrophs tend to have rapid life cycles, higher mutation rates, and expeditious nutrient acquisition systems by means of passive or active transport systems. Indeed, the majority of photosynthetic life-forms are small.

By way of an explicit adaptationist model, consider multilevel selection (MLS) theory, which characterizes ‘individuality’ during the evolutionary transition from a unicellular ancestor to a multicellular descendent. It identifies two stages—an alignment-of-fitness phase (denoted as MLS1) in which the genetic similarity among adjoining cells prevents cell–cell conflict, and an export-of-fitness phase (MLS2) in which cells become so interdependent that fitness (and individuality) must be assigned to the cellular ‘colony’ rather than to each cell (see Folse and Roughgarden, 2010). As noted, MLS1 is achieved by a unicellular bottleneck that assures, at least for a time, the cells share the same genome. MLS2 requires that selection shifts from the level of individual cells to the level of the multicellular entity (Box 1). The key difference between MLS1 and MLS2 is that the fitness of a colony is an additive function of the fitness of individual cells, whereas the fitness of the multicellular descendant is non-additive.

However as noted, MLS1 is not the result of adaptive evolution *per se* (see Niklas and Kutschera, 2010; Niklas and Newman, 2013). Similarly, the adaptive nature of MLS2 is unclear because most eukaryotes lack the soma–germline dyad (i.e. in plants, somatic embryogenesis is the norm). Further, polyclonality is not inconsistent with either viability or individuality, and may be favored in some environments (Bourrat, 2015; Hamant *et al.*, 2019). Nevertheless, phyletic analyses of some lineages are consistent with the MLS1–MLS2 model. Lineages characterized by species with clonal group formation are more likely to have undergone an evolutionary transition to obligate multicellularity than lineages characterized by species with non-clonal group formation (e.g. Fisher *et al.*, 2013), and experimental data from asco- and basidiomycete heterokaryotic fungi reveal competition among genetically different nuclei sharing the same cytoplasm (Jinks, 1952; Davis, 1960).

## Inherent material properties in the transition to multicellularity and IMOs

**Fig. 1.**
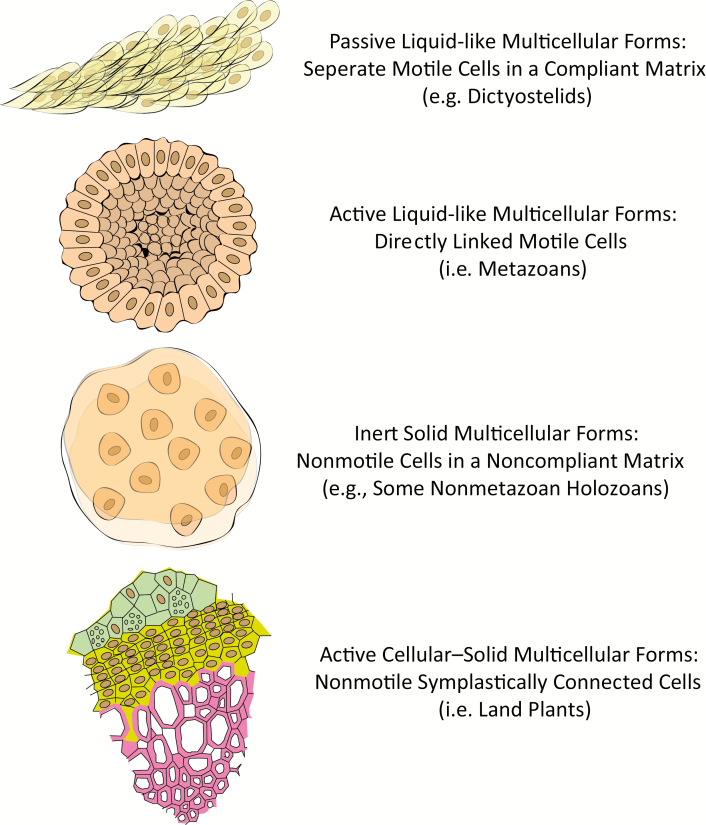
Material properties and characteristics of tissues in multicellular organisms

Because the characteristic physiological genomics and biochemistry of the various IMO clades differ widely, it is not surprising that, as with the means of cell–cell attachment, the bases of intercellular signaling and developmental transitions are divergent at the molecular level (Fig. 1). What is increasingly recognized, however, is that the material properties of these organisms provide inherent morphogenetic propensities and constraints that lead to signature motifs within each of these groups despite significant evolutionary drift at the genetic level. Recent work has also shown that *de novo* self-organization (in contrast to the inheritance of a prior organized state) can obtain functional cell-like features (e.g. Cheng and Ferrell, 2019).

Consider the social amoebozoans, such as dictyostelids. These organisms exhibit a conditional form of IMO by forming collectively motile and structurally differentiated forms at certain phases of their life cycles. In *Dictyostelium discoideum* and related forms, a subpopulation of solitary amoebae signal one another when food is depleted and form liquid-like streams flowing toward biochemically oscillatory signaling centers, culminating in a mound of cells, which topple over, forming a multicellular migratory slug whose movement is driven by internal cell fluxes. The slug then forms a fruiting body (Bonner, 2009). Dictyostelid amoebae behave like cohesive liquids (when streaming, when they are confined within migrating slugs, and when forming fruiting bodies) due to their being embedded in cellulose-based matrices that are compliant and permissive to cell rearrangement (Huber and O’Day, 2017). Cellular mobility within cohesive masses is also promoted by chemotactic responses to extracellular signals (Tan and Chiam, 2014). The cells are also polarized as they migrate individually and collectively (Manahan *et al.*, 2004), but since transient polarization is not consolidated as in developing plant and animal tissues (Niklas *et al.*, 2019), architectural motifs like buds and branches, or lumens and appendages do not form.

The metazoans, or animals, a lineage of multicellular holozoans (non-fungal opisthokonts; reviewed in (Newman, 2016*b*) is the other major group of IMOs whose coherence and functional integration depend on liquid-like properties. In contrast to the dictyostelids, cell–cell attachment in all animal phyla is mediated by transmembrane cell adhesion molecules (cadherins) that coordinate attachment and cytoskeletally driven motility, permitting cells to remain tightly bound to their neighbors while moving past one another (reviewed in Newman, 2016*a*). A consequence of the liquid-like properties of these tissues (as with dictypstelid stages) is that they exhibit a surface tension that causes them to assume the lowest surface-to-volume ratio consistent with the shape of their cellular subunits. This will be a sphere if the subunits are isotropic but will be elongated if the cells become polarized in shape (termed planar cell polarization) and intercalate among one another (reviewed in Newman, 2016*a*; Niklas *et al.*, 2019).

In contrast, polarization of surface properties of individual cells, termed apicobasal (A/B) polarization (reviewed in Newman, 2016*a*; Niklas *et al.*, 2019) has the effect of adding interior spaces or lumens to the repertoire of metazoan forms, since tissues containing A/B polarized cells can undergo an internal sorting process so that the less adhesive portions of cell surfaces come to define interior cavities (Forgacs and Newman, 2005). Just as non-living liquids (e.g. oil and water) can phase separate from each other due to different cohesive properties, so can masses of animal cells with different amounts or types of cadherins on their surfaces. This may explain the origin of the characteristic tissue layering (e.g. gastrulation) seen in all complex animal body plans. None of these things occur in the dictyostelids, where aggregate fluidity is matrix-based and not prone to phase separation, and cell polarity is more transient.

Animal cells can produce extracellular matrices (ECM) of their own, but these usually abrogate the liquid-like properties of the corresponding tissues and can lead to the formation of internal substrata (basal laminae) and stiff exo- and endoskeletons, promoting elastic sheet-like behaviors, all of which contribute to morphological complexity of the metazoans. In no case does the multicellularity of a metazoan species depend solely on such non-liquid modes (reviewed in Newman, 2016*a*). This is in sharp contrast to their sister clades within the holozoans (e.g. Choanoflagellatea, Filasterea, and Ichthyosporea), the multicellular forms of which cannot be counted as IMOs since their integrative properties and morphogenetic behaviors are severely limited by their non-compliant modes of cell–cell attachment (Dayel *et al.*, 2011; Sebé-Pedrós *et al.*, 2013; de Mendoza *et al.*, 2015; reviewed in Newman 2019*a*).

The cells of the Fungi (a sister clade to the holozoans in Opisthokonta) and the Chlorobionta usually have stiff cell walls. They have escaped the dead-end fates of the non-metazoan holozoans. However, to become IMOs via long-range cell–cell communication and morphogenetic functionalities, unlike the metazoans and dictyostelids, plants behave as cellular-solids. The tissues of the multicellular members of these clades are consequently deformable and enzymatically meltable solids that can develop into highly complex forms in ways that are entirely different from those of multicellular organisms with wall-less, motile cells (Hernández-Hernández *et al.*, 2012; Benítez *et al.*, 2018).

The material composition and mechanical behavior of the land plants are best described as a cellular-solid, which is technically defined as a material composed of a solid phase (e.g. the cell wall) and a liquid phase (either a liquid or gas, e.g. protoplasm and air, respectively) (Niklas and Spatz, 2012). When the bulk of the material is composed of a liquid, such as protoplasm, the material behaves as a hydrostatic device (e.g. turgor pressure results in tensile stresses within cell walls thereby establishing the bulk elastic modulus of a tissue). As the volume fraction of a solid phase increases (e.g. either as a result of thickening cell walls, or the loss of protoplasm), the mechanical properties of a cellular-solid shifts from that of a hydrostat to a rigid solid whose elastic modulus depends on the material properties of the solid phase regardless of the fluid’s pressure.

The mechanics of a cellular-solid helps to explain why the evolution of sclerenchymatous tissues permitted plants to rely less on turgor (and thus less on a continuous access to liquid water) for the mechanical support of aerial organs. Note, however, that the evolutionary shift from a unicellular to a multicellular body plan does not necessarily result in a complimentary shift from a hydrostat to a solid. In the context of the land plants, multicellularity can be achieved regardless of the volume fractions of the solid and fluid phases. Land plant multicellularity did result in the appearance of composite cellular-solids—the appearance of different tissues, each with different material properties as a result of different solid and fluid volume fractions. An important feature is the capacity to control the material properties of the solid and fluid phases within living tissues. Metabolic control of the cell wall’s material properties and the viscoelasticity of protoplasm enables sophisticated control of the material properties of each cell or tissue type. Other distinctions between land plant and animal multicellularity are the absence of cell migration (and thus metastasis) owing to non-fluid cell to cell adhesion, meristematic growth and the production of post-embryonic organs, the absence of a germ cell line, the presence of plasmodesmata joining the cytoplasm of all living cells into a continuous symplastic cytoplasm, and the presence of a dyadic life cycle (i.e. the alternation of generations).

These examples show that the inherent morphological motifs of multicellular entities depend on their material properties and their capacity to mobilize the specific physical forces and effects characteristic of those materials (Fig. 1). The genetic repertoires and degree of phylogenetic affinity may be secondary. This is exemplified by the volvocine algae. Initially their cells are attached by numerous cytoplasmic bridges, the result of incomplete cytokinesis (Hoops *et al.*, 2005). But unlike other archaeplastids, their cells do not become cemented together by rigid cell walls, and instead have a flexible glycocalyx, enabling dynamical, polar cell shape changes. These physical properties allow cell sheets to undergo inversion and form hollow, folded structures, similarly to metazoan gastrulation (Höhn and Hallmann, 2011; Matt and Umen, 2016; Niklas *et al.*, 2019). The volvocines represent an evolutionarily derived rather than primordial form of multicellularity, but their deviation from the morphogenetic behaviors of their direct algal ancestors and assumption of animal-type behaviors is related to shared material properties with the latter rather than genetic affinity with the former.

## Conclusions

Multicellularity evolved multiple times in phyletically disparate lineages as a consequence of the physical phenomenon of cell–cell adhesion. Whether or not multicellular forms progressed to become IMOs, in which multicellularity became an obligatory step in a life cycle or developmental program rather than a mere condition-based modality, depended on the integration of adhesion and other physical effects such as diffusion of cell–cell signaling molecules with polarity and other ancestral cell physiological and genomic components (e.g. Young *et al.*, 2011; Olson and Nedelcu, 2016; Grochau-Wright *et al.*, 2017; Elhai and Khudyakov, 2018; Niklas *et al.*, 2019). This integration lead the resulting forms to embody new material properties characterized by ‘dynamical patterning modules’, physical forces and effects mobilized by the materials’ gene products and other molecules (Newman and Bhat, 2009; Benítez *et al.*, 2018; Newman and Niklas, 2018).

In all cases, the physical mode of cell–cell attachment or association is key to the ability of multicellular entities to progress to IMOs, that is, to exhibit ‘evolvability’. In dictyostelids this was a fluid matrix permissive to cell signaling and motility, in animals a unique form of extracellular–intracellular linkage that turned the aggregate itself into a chemically excitable fluid, and in plants and fungi, most remarkably, solid cementing materials that are meltable, deformable, and permissive to long-range cell–cell communication of both direct and indirect natures. Multicellular of evolvability is, therefore, not simply a property or capacity of the genome (Wagner and Altenberg, 1996), but rather the ‘processes that build phenotypic structures through reiterated feedback interactions between genes, cells, and tissue aggregates, which include non-genetic elements such as physics, function, or environmental factors’ (Müller, 2019).

We suggest, therefore, that the search for ‘canonical’ ancient gene regulatory networks underlying the origination of multicellularity is futile. Moreover, while three phases can be identified for the emergence of multicellularity in each lineage; i.e. origination by widely different cell–cell adhesion modalities, integration by lineage-specific physiological mechanisms, and autonomization by natural selection (Müller and Newman, 1999), the first of these was not *a priori* a consequence of natural selection, but could have had a variety of causes unrelated to adaptation. Once a novel living material was thus constituted, however, the other two phases would have followed to the degree that the material’s inherent properties facilitated new modes of innovation, adaptation, and survival.
